# Malignant Solitary Fibrous Tumor of the Inguinal Region: A Case Report

**DOI:** 10.7759/cureus.47123

**Published:** 2023-10-16

**Authors:** Fatema Bunajem, Tareq Al Taei, Nawraa Mujbel, Ahmed Al Shaikh, Sarah Al Mail

**Affiliations:** 1 Radiology, Salmaniya Medical Complex, Manama, BHR; 2 Radiology, Salmaniya Medical Complex, Busaiteen, BHR; 3 Internal Medicine, Salmaniya Medical Complex, Manama, BHR

**Keywords:** spindle cell sarcoma, malignant solitary fibrous tumor, magnetic resonance imaging (mri), computed tomography (ct), solitary fibrous tumor (sft)

## Abstract

A solitary fibrous tumor (SFT) is a rare spindle cell neoplasm in adults, usually found in the pleural and thoracic cavities. We report an interesting case of a malignant solitary fibrous tumor in a 64-year-old male who presented with a history of swelling in his right inguinal region that gradually increased in size during the past three years. Computed tomography (CT) and magnetic resonance imaging (MRI) scans of the abdomen and pelvis showed a rounded solid mass originating from the right inguinal canal suggestive of sarcoma. Elective excision of the mass was done under general anesthesia with histopathology confirming the diagnosis of high-grade spindle cell sarcoma in keeping with a malignant solitary fibrous tumor. Postoperatively, the patient had no complications and was discharged on postoperative day 4. The patient was then treated with radiotherapy. He remained free of recurrence for two years postoperatively.

## Introduction

A solitary fibrous tumor (SFT) is an uncommon spindle cell neoplasm in adults [1]. The reported incidence accounts for less than 2% of all soft tissue tumors and less than 1% of all adult malignancies [2,3]. Initially, it was found in the pleural and intrathoracic cavities. However, it could occur in different parts of the body [1]. SFTs are usually benign in behavior but slow-growing tumors, and malignant SFT subtypes have been discussed in a few pieces of literature [4]. Available data on the imaging findings are nonspecific due to disease rarity, which makes reaching an accurate diagnosis preoperatively challenging [5]. In addition, it necessitates the use of tissue biopsy for histopathology and immunohistochemistry analysis. Herein, we report a case of a solitary fibrous tumor of the right inguinal region in a middle-aged male.

## Case presentation

A 64-year-old male, with an unremarkable medical history, presented to the surgery outpatient department reporting a swelling in his right inguinal region that had gradually enlarged in size during the past three years. The patient complained of a new onset of pain in the area. The patient denied any history of fever, weight loss, and gastroenterology or genitourinary symptoms.

**Figure 1:**
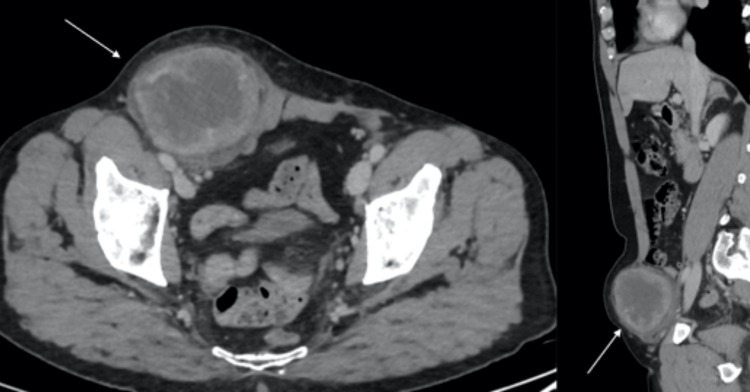
Axial and sagittal contrast-enhanced computed tomography scan of the abdomen and pelvis showing a right inguinal lesion (white arrows)

Physical examination revealed a 7 × 9 cm firm, nonmobile, and non-tender right-sided groin swelling with no inflammatory signs on the skin overlying the swelling. Manual reduction was attempted but was unsuccessful. Full blood tests were ordered, including tumor markers (cancer antigen (CA) 15-3 and CA 19-9) (Table 1). However, the laboratory values were unremarkable. The patient also underwent a radiological examination in the form of contrast-enhanced computed tomography (CT) scan of the chest, abdomen, and pelvis, followed by abdominal magnetic resonance imaging (MRI) for further characterization. Contrast-enhanced CT scan of the abdomen and pelvis showed a rounded, well-defined solid mass originating from the right inguinal canal (Figure 1). It has heterogeneous enhancement with surrounding soft tissue fatty stranding. There was no intra-lesion calcification and no regional lymphadenopathy with no invasion of the nearby blood vessels. Contrast-enhanced CT scan of the chest was also performed to rule out possible distal disease metastasis, which showed no definitive intrapulmonary masses.

**Table 1:** Laboratory investigations and their values CA: cancer antigen

Laboratory test	Value	Unit	Reference range
White blood cell count	11	×10^9^/L	3.6-9.6
Hemoglobin	14.2	g/dL	12-14.5
Platelet	279	×10^9^/L	150-400
CA 15-3	28.3	U/mL	<31.3
CA 19-9	17.4	U/mL	<37
Random blood glucose	4.3	mmol/L	3.6-8.9
Urea	6.4	mmol/L	3.2-8.2
Creatinine	97	µmol/L	53-97

**Figure 2:**
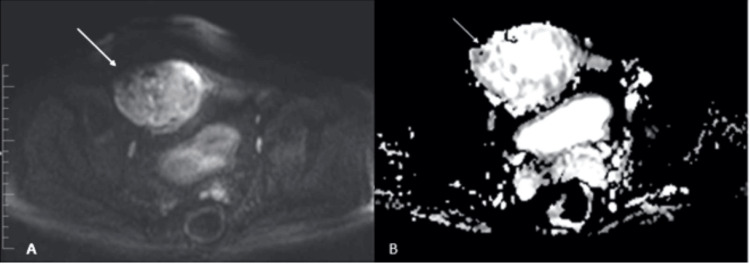
Axial diffusion (A) and ADC (B) magnetic resonance imaging showing patchy restricted diffusion also noted in the periphery of the non-necrotic region (white arrows) ADC: apparent diffusion coefficient

**Figure 3:**
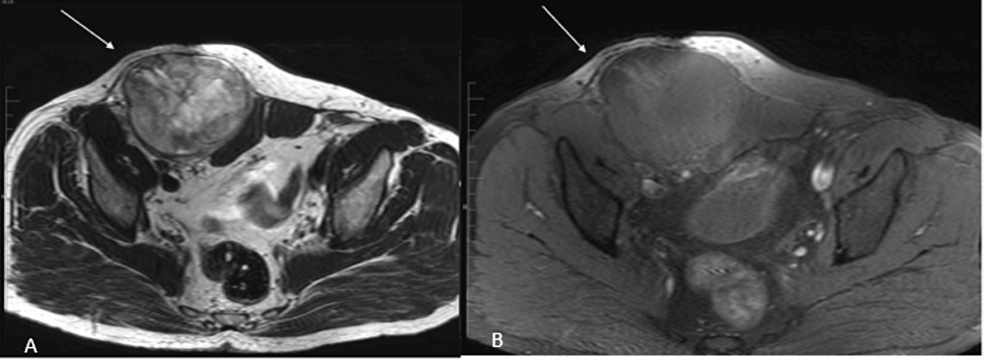
Axial T2-weighted images (A) and STIR (B) magnetic resonance imaging of the pelvis showing a right inguinal lesion (white arrows) STIR: short-TI inversion recovery

**Figure 4:**
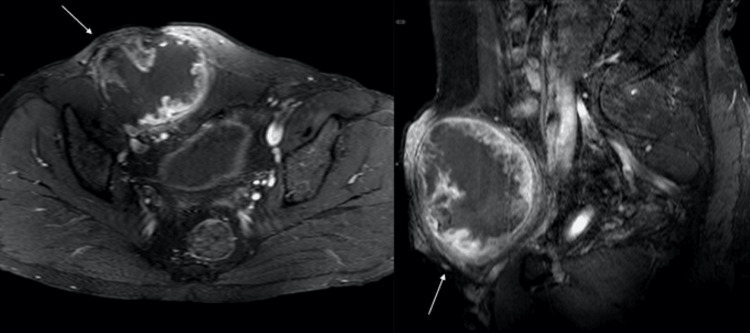
Axial and sagittal T1 fat suppression post-contrast showing heterogeneous enhancement with a large area of non-enhancing necrosis (white arrows)

Abdominal MRI result re-demonstrated the previously described lobulated altered signal intensity soft tissue mass lesion in the right inguinal region measuring 7.2 × 8.5 × 9.8 cm in craniocaudal (CC) × anteroposterior (AP) × transverse (TR) dimensions. The lesion appeared hypointense on T1-weighted (T1W) images, hyperintense on T2-weighted (T2W) images, and not suppressed on short-TI inversion recovery (STIR) sequence with heterogeneous enhancement on post-contrast images and a large area of non-enhancing necrosis. Patchy restricted diffusion was also noted in the periphery of the non-necrotic region (Figures 2-4). These findings were suggestive of sarcoma and less likely to be metastasis disease.

**Figure 5:**
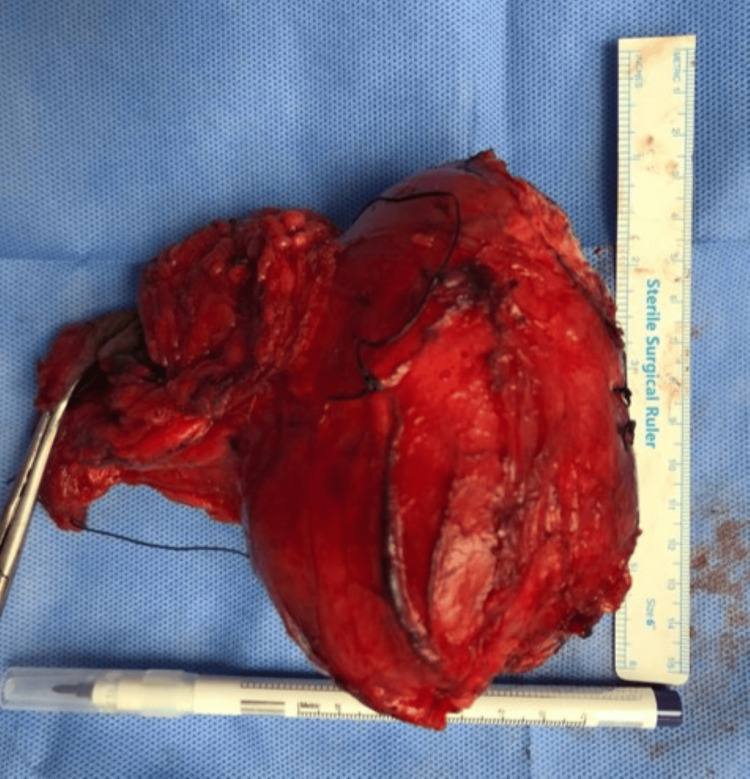
Gross specimen showing an encapsulated right groin mass

The patient underwent elective excision of the right groin mass under general anesthesia. Surgical intervention revealed the presence of a round, well-encapsulated right groin mass with some adherence to adjacent structures and within the inguinal canal (Figure 5). The postoperative course was uneventful, and the patient was discharged on the fourth postoperative day.

**Figure 6:**
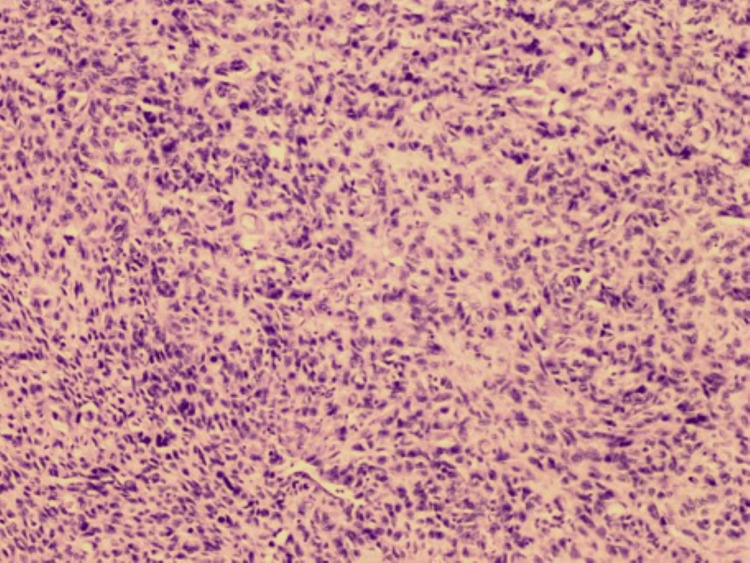
H&E, original magnification ×100, showing sheets of malignant cells with nuclear pleomorphism and hyperchromasia H&E: hematoxylin and eosin stain

**Figure 7:**
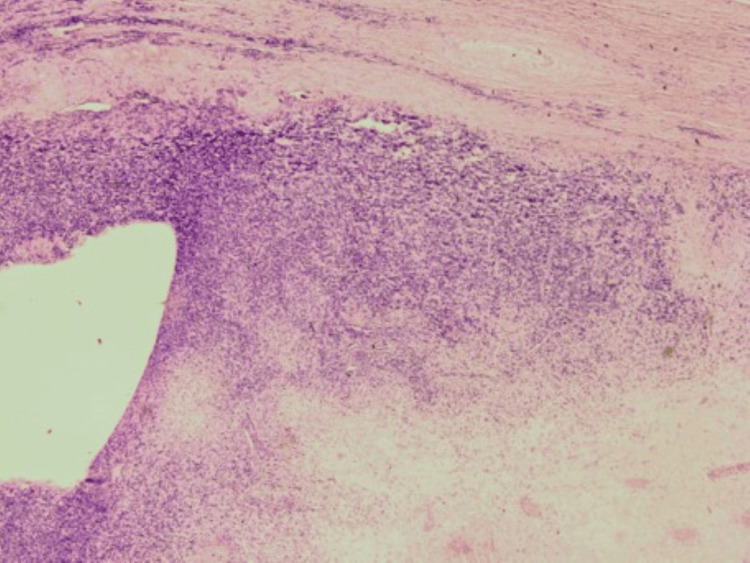
H&E, original magnification ×20, showing geographic tumor necrosis with hypercellular area of malignant spindle cells H&E: hematoxylin and eosin stain

**Figure 8:**
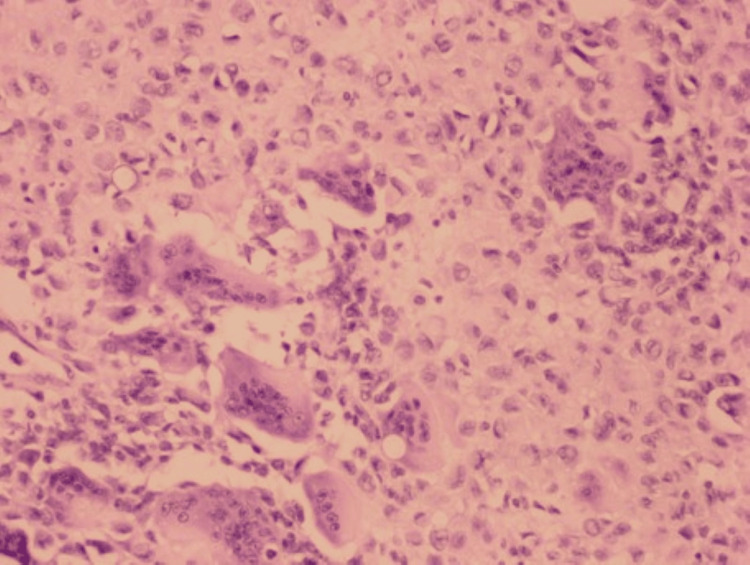
H&E, original magnification ×20, showing geographic tumor necrosis with hypercellular area of malignant spindle cells H&E: hematoxylin and eosin stain

On gross examination, the tumor was solitary and located below the subcutaneous fatty tissue, measuring 12 × 9 × 6.5 cm. A cut section of the tumor showed central hemorrhage, necrosis, and yellowish areas. Microscopic examination showed alternating hypocellular and hypercellular areas of spindle cells with mild to moderate pleomorphism, a large area of necrosis, and a high mitotic rate of approximately 16 mitoses per 10 high-power fields. The hypercellular area showed a hemangiopericytoma-like pattern with large gaping and staghorn-like vessels. Immunohistochemically, the tumor stained positive for cluster of differentiation 34 (CD34) and CD99 only with all other immunohistochemistry in neoplastic cells beginning negative (S100, human melanoma black 45 (HMB45), B-cell lymphoma 2 (BCL2), CD31, AE/AE3, cytokeratin 7 (CK7), 34BetaE12, spinal muscular atrophy (SMA), muscle-specific actin (MSA), Desmin, myogenic differentiation 1 (MyoD1), and p63). Additionally, fluorescence in situ hybridization (FISH) for mouse double minute 2 homolog (MDM2) gene amplification was performed and was negative. The pathological diagnosis was high-grade spindle cell sarcoma with features suggestive of a malignant solitary fibrous tumor (Figures 6-8).

The patient has been sent for radiotherapy with a close follow-up plan to rule out disease recurrence. He remained free of recurrence for two years postoperatively.

## Discussion

A solitary fibrous tumor was first described by Klemperer and Rabin in 1931, who stated that they are rare soft tissue tumors that mostly arise from mesenchymal cells and are more common to originate in the pleura of the lung [6]. However, other locations have been documented recently, including the head and neck, extremities, and abdominal organs [5]. Only a limited number of cases have been described in the medical literature, which reports inguinal location [5]. In a 10-year retrospective study done in China, only five cases of SFTs seen in the inguinal area were identified in the literature [7].

The etiology of SFT occurrence in the body is poorly understood, and no risk factors associated with disease development have been identified. There is no gender predilection, both males and females can be equally affected, and it is mostly seen in the fifth or sixth decade of adult life [8].

The clinical features of SFTs are slow-growing mass that is usually painless with the possibility of causing a mass effect on adjacent structures. Rarely, they can present as part of paraneoplastic syndromes, in which the tumor secretes insulin-like growth factor [9]. In our case, the blood glucose test result was within the normal range.

The role of imaging in the diagnosis of SFTs is limited due to nonspecific imaging findings, but it is crucial to help characterize the tumor and narrow the differentials. Generally, on non-enhanced CT scans, the attenuation value varies depending on the content of the lesion. Moreover, evidence of lesion calcification is uncommon but could raise the possibility of a malignant mass. In contrast to enhanced CT scans, different degrees of enhancement reflect the degree of tumor cellularity and vascularity. About 100% of malignant and 60% of benign lesions reported show heterogeneous enhancement. For superficial SFTs that can be found in the extremities or the head and neck, an ultrasound examination could be used as an initial study. However, it lacks specificity as well. SFT findings on MRI show a hypointense signal on T1W images with variable signal intensity on T2W images. They are usually highly vascular, which may show intense heterogeneous enhancement in post-contrast images. Positron emission tomography/CT (PET/CT) scanning is important in recognizing disease recurrence or distal metastases [10].

For accurate diagnosis, pathology evaluation is crucial. Microscopically, SFTs are composed of spindle cells of variable cellularity that have thin-walled, large, branching, staghorn-like blood vessels within their stroma [11]. According to the World Health Organization’s classification of soft tissue tumors, criteria that support malignant SFTs include high tissue cellularity, evidence of more than four mitoses per 10 high-power fields, tumor necrosis, and infiltrative borders [4]. In addition, immunohistochemistry markers such as CD34, CD99, and BCL-2 are the most sensitive and specific in the establishment of the diagnosis [11].

The primary treatment for both benign and malignant SFTs is surgical resection with the possibility of using radiotherapy or chemotherapy as adjuvant treatment to decrease the risk of disease recurrence or metastasis. There is no standard treatment for SFTs. However, a precise management plan by a multidisciplinary team is recommended [11].

## Conclusions

Solitary fibrous tumors of the inguinal region are very rare. As a result, it could contribute to misdiagnosis and subsequently delay in management. The available imaging features are nonspecific, which makes the diagnosis challenging. However, a good knowledge of this disease entity is required to build a proper differential diagnosis. Furthermore, a comprehensive gathering of clinical, radiological, and histopathological data is needed to reach an accurate diagnosis.
